# TRAF6 promotes chemoresistance to paclitaxel of triple negative breast cancer via regulating PKM2‐mediated glycolysis

**DOI:** 10.1002/cam4.6552

**Published:** 2023-09-25

**Authors:** Han Xu, Longzhi Li, Bing Dong, Ji Lu, Kun Zhou, Xiaoxing Yin, Huizhen Sun

**Affiliations:** ^1^ Department of General Surgery Jing'an District Center Hospital of Shanghai Shanghai China; ^2^ Department of Obstetrics and Gynecology Xinhua Hospital Affiliated to Shanghai Jiaotong University School of Medicine Shanghai China

**Keywords:** breast cancer, chemoresistance, glycolysis, PKM2, TRAF6

## Abstract

Ample evidence reveals that glycolysis is crucial to tumor progression; however, the underlying mechanism of its drug resistance is still worth being further explored. TRAF6, an E3 ubiquitin ligase, is well recognized to overexpress in various types of cancer, which predicts a poor prognosis. In our study, we discovered that TRAF6 was expressed more significantly in the case of triple‐negative breast cancer (TNBC) than in other of breast cancers, promoting chemoresistance to paclitaxel; that inhibited TRAF6 expression in the chemoresistant TNBC (TNBC‐CR) cells enhanced the sensitivity by decreasing glucose uptake and lactate production; that TRAF6 regulated glycolysis and facilitated chemoresistance via binding directly to PKM2; and that overexpressing PKM2 in the TNBC‐CR cells with TRAF6 knocked down regained significantly TRAF6‐dependent drug resistance and glycolysis. Additionally, we verified that TRAF6 could facilitate PKM2‐mediated glycolysis and chemoresistance in animal models and clinical tumor tissues. Thus, we identified the novel function of TRAF6 to promote glycolysis and drug resistance in TNBC with the regulation of PKM2, which could provide a potential molecular target for TNBC treatment.

## 1 INTRODUCTION

In breast cancer, which is the most frequent malignancy in women,^1^ triple‐negative breast cancer (TNBC), detected as negative expression of estrogen receptor (ER), progesterone receptor (PR), and epidermal growth factor receptor 2 receptor (HER2) by immunohistochemistry, is easy to relapse and metastasize.^2^ Given a lack of effective endocrinotherapy and molecular targeted therapy, the main treatment option is chemotherapy for TNBC.^3^, ^4^ However, TNBC patients usually develop chemotherapy resistance, resulting in a poor prognosis.^2^, ^5^ Paclitaxel, routinely applied to breast cancer, was reported to cause mitosis to cease and cell death by stabilizing microtubules and inhibiting their decomposition.^6^ Despite the therapeutic benefits paclitaxel possesses, half of TNBC patients could become resistant to the chemotherapy after 0.5–1 year of finishing treatment.^7^ Hence, it is imperative that the mechanism of drug resistance in TNBC be investigated as the key approach to improving the patient's prognosis.

Tumor necrosis factor receptor‐associated factor 6 (TRAF6), as an E3 ubiquitin ligase,^8^ has a significant function in cancer biology processes by triggering cell signaling pathways, including, the NF‐κB signaling pathway,^9^ the MAPK signaling pathway,^10^ and the Wnt/β‐catenin signaling pathway.^11^ TRAF6 has been confirmed recently to be a prognostic biomarker that is likely to overexpress in such cancers as pancreatic cancer,^9^ renal cell carcinoma,^12^ and colorectal cancer.^13^ TRAF6 was reported to facilitate the occurrence and progression of tumors involved in proliferation, apoptosis, and invasion.^14^ All this indicates that since TRAF6 regulation could facilitate the modulation of tumor progression of various types, much importance should be attached to the exploration of its exact impact on TNBC chemoresistance.

Cancer cells are inclined to uptake more glucose than normal cells to support aerobic glycolysis, even in a well‐oxygenated environment, as the Warburg effect.^15^, ^16^, ^17^ Accumulating evidence suggests that elevated aerobic glycolysis means a growth advantage to tumor cells, promoting resistance to chemotherapeutics by providing energy and metabolic intermediates.^18^, ^19^, ^20^ When upregulated widely in tumor cells, pyruvate kinase M2 (PKM2) is involved in cancer metabolism and tumor growth.^21^, ^22^ The breast cancer cells that express PKM2 tend to show glycolysis adaptation, which facilitates their ability to maintain metastasis capacity.^23^ Additionally, previous studies have indicated that inhibiting PKM2 could increase sensitivity to chemotherapeutics in multiple tumors, such as colorectal cancers,^24^ bladder cancers,^25^ and multiple myeloma cells.^26^ We thus hypothesized that PKM2 could regulate paclitaxel resistance in TNBC by altering glycolysis.

In our study, we discovered that TRAF6, which was overexpressed in TNBC patient‐derived specimens, was related to chemoresistance; that glycolysis activity was measured to be promoted in chemoresistant TNBC cells; that the inhibited expression of TRAF6 enhanced the sensitivity of TNBC to paclitaxel; and that TRAF6 was capable of binding to PKM2 directly to promote TNBC cells glycolysis, which increased resistance to paclitaxel in vitro and vivo. From our research, therefore, a potential prognosis molecule could be pursued as a treatment target for TNBC.

## 2 MATERIALS AND METHODS

### 2.1 Patients and clinical tissue specimens

A total of 185 invasive ductal cancer tissue samples from TNBC patients were obtained from Jing'an District Central Hospital of Shanghai with approval from the ethics committee and informed consent from the patients for the experiment. As indicated in Table S1, a summary was made of the detailed clinicopathological features.

### 2.2 Cell culture, lentivirus preparation, and cell transfection

From Fudan University Cancer Institute were obtained MDA‐MB‐231HM cells (originated from MDA‐MB‐231 cells with high lung metastasis, simplified as 231HM cells), and from ATCC were obtained MDA‐MB‐231 cells, simplified as 231 cells, which all were cultured with Dulbecco's modified Eagle's medium (DMEM), added with 10% fetal bovine serum (FBS) and 100 U/mL penicillin, in a temperature of 37°C and humidified environment of 5% CO_2_. The plasmids, which carried TRAF6 shRNA1 (5′‐GAGAACACCCAGTCACACA‐3′), TRAF6 shRNA2 (5′‐GCCACGGGAAATATGTAATAT‐3′), and PKM2 full‐length cDNA, were transfected into 293T cells, respectively, before being cultured with fresh medium after 8 h of transfection. When the supernatant with lentivirus was harvested 48 h later, the cell debris was removed with a 0.45 μm filter. These supernatant viruses were used to infect the corresponding cells to generate TRAF6 knockdown or PKM2 overexpression cells. The cells were then screened with 5 μg/mL puromycin for a period of 7–10 days; consequently, the expressions of TRAF6 and PKM2 were examined by western blotting.

### 2.3 Plate colony formation, cell viability, and cell apoptosis assay

In six‐well plates, 700 cells were seeded per well. Twenty‐four hours later, the cells were cultured with 15 μg/mL paclitaxel in complete medium (DMEM containing 10% FBS) for 2 weeks. Subsequently, cells were washed gently with PBS and fixed with 4% paraformaldehyde for 20 min at room temperature (E672002; Sangon Biotech). Finally, cells were stained with crystal violet (C0121; Beyotime). Under the microscope, the number of colonies containing 50 cells was calculated, as was plate colony formation efficiency based on the formula: plate colony formation efficiency = (number of colonies/number of cells inoculated) × 100%.

The CCK‐8 kit (Dojindo Laboratory) was used to detect cell viability. In each well of 96‐well cell culture plates were inoculated 5000 cells. When adhered, the cells were then cultured in various concentrations of paclitaxel diluted with complete medium. The paclitaxel solution was sucked up 48 h later, with the dead cells cleaned with PBS, followed by an addition of 100 μL of 5% CCK‐8 to each well, which was prepared in the medium of 10% FBS, before being incubated in the incubator for 2 h. In the 96‐well plate was absorbed 95 μL liquid, so that the OD value was calculated at 450 nm. According to different OD values of different drug concentrations, the fitting curves were drawn: cell viability (% of control) = {OD value (experimental group) − OD value (blank group)}/{OD value (control group) − OD value (blank group)}. IC50 was presented on the software Graphpad Prism 9.0 via cell viability.

Paclitaxel‐induced cell apoptosis was investigated via flow cytometry (Beckman Coulter) after the staining of propidium iodide (PI) and Annexin V. 1 × 10^6^ cells were seeded in each 60 mm dish to be kept for 24 h, followed by the treatment of paclitaxel (40 μg/mL) for 24 h, before being trypsinized and washed twice in phosphate‐buffered saline (PBS). In 100 mL of binding buffer, 1 × 10^5^ cells were resuspended and added to 5 mL of 2 mg/mL Annexin V and 5 mL of 50 mg/mL PI. The cells were examined by flow cytometry after being incubated in the dark for 15 min.

### 2.4 qRT‐PCR array

When total RNA was extracted from the cells by the FastPure Cell/Tissue Total RNA Isolation Kit (Vazyme) in accordance with the operating instructions, the reverse transcription of mRNA was performed with PrimeScriptTM RT Master Mix (Takara). The RNA level was investigated by qRT‐PCR with SYBR Premix Ex Taq (Takara), and 18S RNA was taken as the endogenous control for mRNA. Fold changes were calculated by relative quantification (2^−ΔΔ*Ct*^).

### 2.5 Western blotting

RIPA lysis was employed to extract total protein, whose quantification was performed with the BCA protein assay kit (Beyotime) and separation with SDS‐PAGE gel. The protein was transferred to PVDF membrane, which was blocked with quick blocking buffer (Beyotime), followed by incubation with specific rabbit antibodies (1:1000; Cell Signaling Technology) at 4°C overnight. Goat anti‐rabbit secondary antibody, which was conjugated to the horseradish peroxidase (1:1000; Cell Signaling Technology), was incubated at room temperature for 1 h. Consequently, the protein was shown with a chemiluminescent horseradish peroxidase substrate (Beyotime) for imaging with the E‐Gel Imager (Bio‐Rad).

### 2.6 Immunohistochemistry assay

In xylene, the tissue sections were deparaffinized, and in a graded series of ethanol, they were rehydrated before being boiled in 0.01 mol/L sodium citrate buffer (pH 6.0) in a water bath kettle at 95°C for 10 min to undergo antigen retrieval. The tissue sections were incubated with an antibody at 4°C overnight in a humid chamber, when the endogenous peroxidase activity was blocked with 0.3% hydrogen peroxide and the nonspecific protein was bound with 1.5% normal goat serum. After that, the antibodies were localized on the tissue sections, which had been incubated with biotinylated goat anti‐mouse or goat anti‐rabbit IgG for 30 min, followed by an analysis on the LSAB system (Dako).

### 2.7 RNA‐sequencing

When extracted from the breast cancer cells, total RNA was treated with mRNA Capture Beads (Vazyme) so that polyA RNA was enriched. Based on the VAHTS mRNA‐seq v2 Library Prep Kit for Illumina (Equitech‐Bio), an RNA library was prepared, and the paired‐end sequencing was done by RiboBio Co., Ltd. with Illumina HiSeq 3000. To perform the analysis of the RNA‐sequencing data, sequencing reads were compared on the spliced‐reads comparator of HISAT2, based on the human genome collection as a reference genome. The gene expression level of each transcript was set as reads per million exon model per kilobase. Gene Set Enrichment Analysis (GSEA) was applied to the annotation of gene function. When the gene expression multiple changes were >2 with a *p* value <0.05 through Cufflinks calculation, the genes were considered to be expressed differentially and significantly.

### 2.8 Immunoprecipitation and mass spectrometry analysis

RIPA lysis was used to extract total protein. Immunoprecipitated magnetic beads were co‐incubated with TRAF6/IgG antibody for 30 min, and TRAF6/IgG antibody combined with magnetic beads was co‐incubated in the medium for 1 h at room temperature for the antigen precipitation reaction. In the cells, consequently, TRAF6 protein was enriched, and the complex was washed on the magnetic rack. Finally, the protein complexes were eluted from the magnetic beads by heating them for 8 min in a metal bath at 95°C. SDS‐PAGE was applied to separate protein complexes, which were analyzed via liquid chromatography/mass spectrometry (LC/MS) and western blotting. The mass spectrometry was performed at Servicebio Company.

### 2.9 Glycolysis assays: glucose uptake, ATP production, lactate release, and extracellular acidification rate

The glucose uptake rate was determined with the Glucose Assay Kit (BioVision); the ATP levels were detected with the ATP Assay Kit (Promega); and the extracellular lactate production was measured with the Lactate Assay Kit (BioVision). All the operations were performed in accordance with the instruction manual. The calculated values were normalized to the protein concentration. The extracellular acidification rate (ECAR) was examined by the Seahorse XF Glycolysis Stress Test Kit (Agilent Technologies) in accordance with the operating instructions in the form of sequential addition to each well with glucose (10 mM), oligomycin (2 mM), and 2‐deoxyglucose (100 mM). The ECAR values were calculated after a standardized cell count and plotted as an average ± SD.

### 2.10 Tissue immunofluorescence technique

In xylene, the paraffin sections were deparaffinized, and in a graded series of ethanol, they were rehydrated before being washed thrice each for 5 min with 0.01 M PBST (PBS containing 0.2% Tween‐20). Followingly, the antigen retrieval of the tissue sections was accomplished in 0.01 mol/L sodium citrate buffer (pH 6.0) within a water bath kettle at 95°C for 15 min. Sealed with 5% donkey serum prepared by PBS for 30 min and cleaned with PBS, the sections were added to 100 μL of primary antibody (all the primary antibodies were administered at 1:1000 dilutions by PBS) to be incubated at 4°C overnight. Afterwards, the sections were washed thrice with PBST before being added with 100 μL fluorescent secondary antibody (all the secondary antibodies at 1:250 dilutions by PBS) for a 1‐h incubation at room temperature and away from light. After that, the sections were cleaned with PBST thrice before being sealed, as in the same case of fluorescence in situ hybridization. The protein co‐localization was observed under confocal laser microscopy.

### 2.11 Tumor xenograft assay

The female nude mice were provided by the SLRC Laboratory Animal Center. The cells of 231, 231‐CR, 231‐CR/TRAF6i, and 231‐CR/TRAF6i/PKM2 were suspended in PBS, from which a 100‐μL cell suspension of a total of 1 × 10^7^ cells were injected subcutaneously into each nude mouse. In the mice, the tumor size was measured, and the body weight was weighed weekly. Three weeks later, paclitaxel (10 mg/kg) was administered through a tail intravenous injection weekly. The mice were reared for 7 weeks after injection until euthanized, and their tumors were removed to be measured in size and weight. The tumor samples were treated according to experimental needs.

### 2.12 Statistical analysis

The results were presented as the group Mean ± SEM on GraphPad Prism 8 software. For two‐group comparisons, a student *t*‐test was applied to analyze the data, with the significant difference statistically considered as *p* < 0.05. Each experiment was repeated thrice.

## 3 RESULTS

### 3.1 Overexpressed TRAF6 to be related with prognosis in TNBC

**FIGURE 1:**
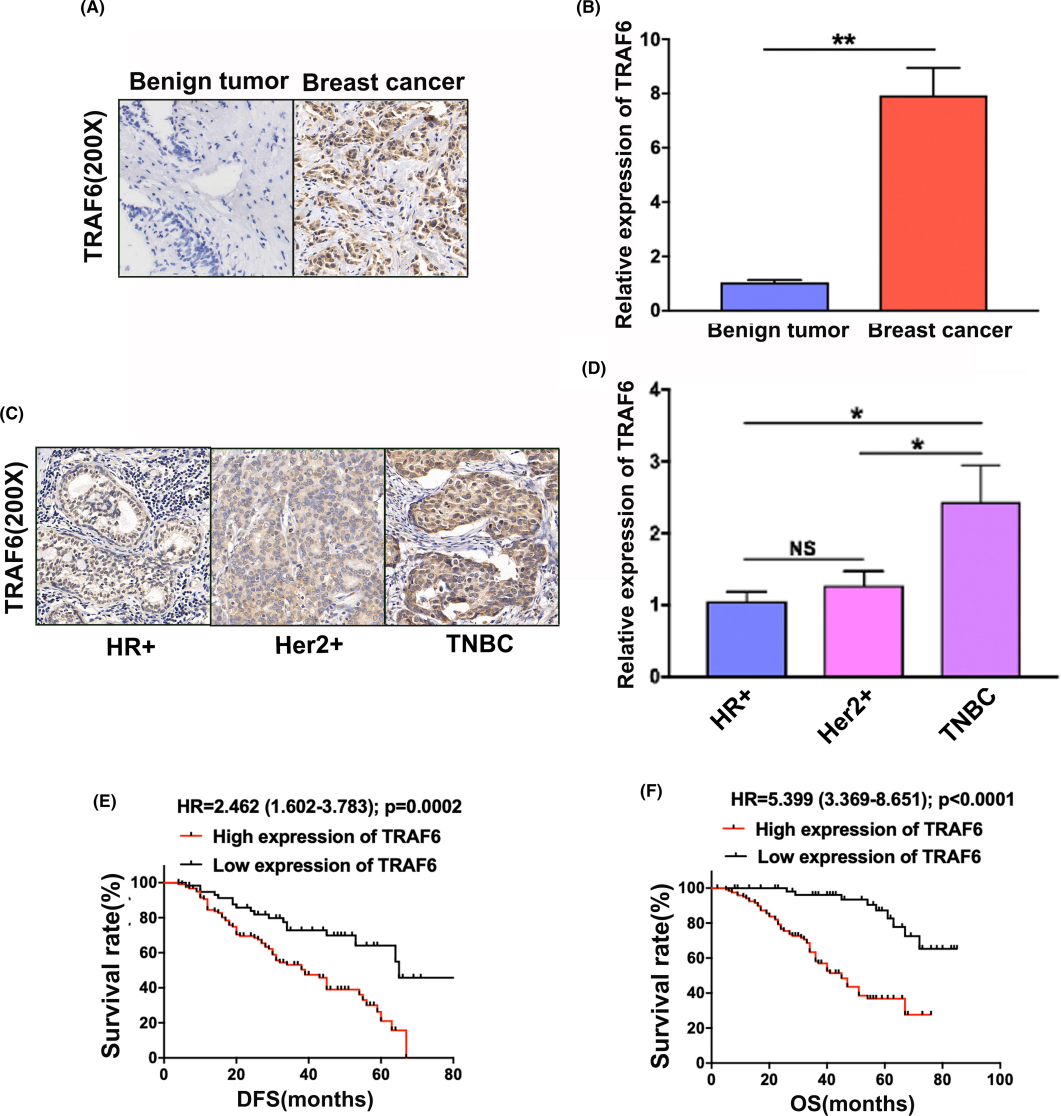
Upregulated TRAF6 in triple‐negative breast cancer (TNBC) to be correlated with poor prognosis. (A) TRAF6 expression detected by immunohistochemistry in breast benign tumor and breast cancer, respectively; (B) TRAF6 mRNA level detected by qRT‐PCR in breast benign tumors and breast cancers, respectively; (C) TRAF6 expression detected by immunohistochemistry in HR+, HER2+ and TNBC, respectively; (D) TRAF6 mRNA level detected by qRT‐PCR in HR+, HER2+ and TNBC, respectively; (E) Kaplan–Meier analysis showing low TRAF6 expression to predict favorable disease‐free survival (DFS) in TNBC patients; (F) Kaplan–Meier analysis showing low TRAF6 expression to predict favorable overall survival (OS) in breast cancer patients among TNBC patients; *p < 0.05, **p < 0.01.

As manifested in the immunohistochemistry, TRAF6 was detected to present overexpression in breast cancer (Figure 1A). Based on qRT‐PCR, the TRAF6 mRNA level was verified to be higher in breast cancer than in benign tumors (Figure 1B). From the analysis of TRAF6 expressions in various molecular subtypes of breast cancer tissues, moreover, TRAF6 protein and mRNA levels were both found to be higher in TNBC than in human epidermal growth factor receptor 2 overexpressed (HER2+) and hormone receptor positive (HR+) breast cancers by qRT‐PCR and immunohistochemistry, respectively (Figure 1C,D). Since TNBC is known to possess a high rate of invasiveness and recurrence, we hypothesized that TRAF6 could have the potential to promote breast cancer progression.

In the immunohistochemical staining, which was conducted in a tissue microarray comprising 185 TNBC specimens to reveal the correlation between TRAF6 expression and TNBC patients prognosis, the analysis of Kaplan–Meier curves indicated that the high expression levels of TRAF6 predicted a lower disease‐free survival rate (Figure 1E) and a slower overall survival rate (Figure 1F).

### 3.2 TRAF6‐promoted paclitaxel resistance in TNBC

**FIGURE 2:**
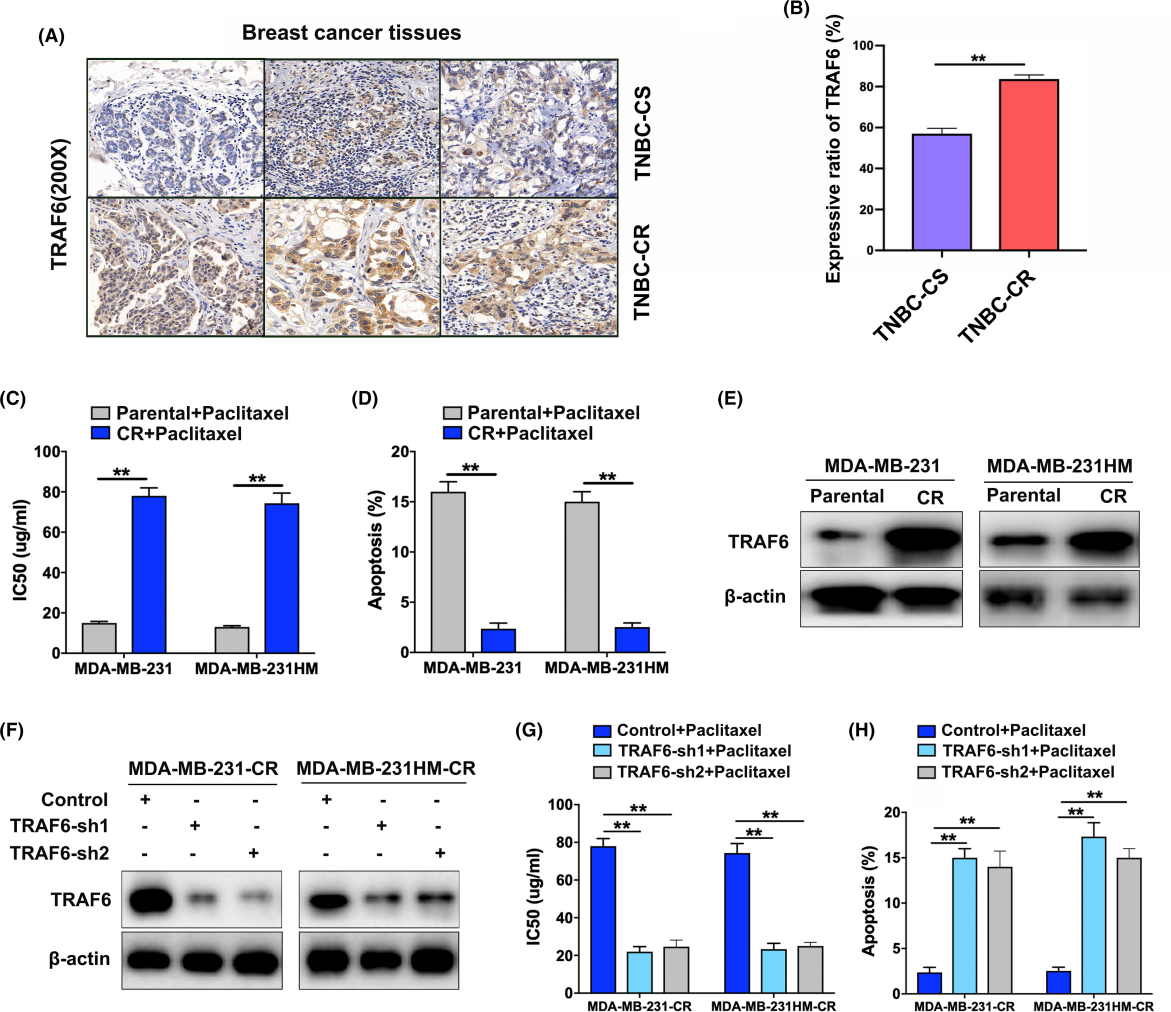
TRAF6‐promoted drug resistance of triple‐negative breast cancer (TNBC) cells. (A) TRAF6 expressions detected by immunohistochemistry, respectively, in TNBC which were sensitive (TNBC‐CS) or resistant (TNBC‐CR) to neoadjuvant chemotherapy; (B) quantitative analysis of TRAF6 expression levels in TNBC‐CS and TNBC‐CR tissues; (C) IC50 values of paclitaxel calculated in TNBC parental cells and chemoresistant cells by CCK‐8; (D) cell apoptosis rate examined by flow cytometry in TNBC parental and chemoresistant cells; (E) western blotting conducted to detect the expression of TRAF6 in TNBC parental and chemoresistant cells; (F) two types of shRNAs introduced to suppress TRAF6 expression, which was confirmed by western blotting; (G) IC50 values of paclitaxel measured in TNBC‐CR cells by CCK‐8 with TRAF6 downregulated; (H) paclitaxel‐induced cell apoptosis ratio analyzed by flow cytometry in TNBC‐CR cells with TRAF6 expression inhibited; **p < 0.01.

In the research on the association of TRAF6 expression with TNBC chemoresistance, we detected the expression in the needle biopsy specimens of TNBC patients who had received neoadjuvant chemotherapy. The data indicated that the expressional level of TRAF6 protein was significantly higher in the TNBC resistant to chemotherapy (TNBC‐CR) than in those sensitive to chemotherapy (TNBC‐CS) by immunohistochemistry (Figure 2A,B). Since paclitaxel is one of the prime chemotherapeutics for TNBC, in the calculation of paclitaxel IC50 to investigate the association of TRAF6 with paclitaxel resistance of TNBC in 231 parental cells, 231 chemoresistant cells (MDA‐MB‐231‐CR or 231‐CR), 231HM parental cells, and 231HM chemoresistant cells (MDA‐MB‐231HM‐CR or 231HM‐CR), respectively (Figure 2C), the flow cytometry analysis showed that the apoptosis ratio was significantly decreased in the chemoresistant cells when compared to the parental ones (Figure 2D). As shown by western blotting, TRAF6 expression was detected to be significantly higher in the chemoresistant cells than in the parental ones (Figure 2E).

According to the investigation of the biological function of TRAF6, in which two shRNAs were constructed to silence TRAF6 expression, the effect of the shRNAs was confirmed by western blotting (Figure 2F). When TRAF6 level was downregulated, cell proliferation was significantly inhibited by the plate cloning experiment (Figure S1A,B), and CCK‐8 assays showed that cell viability was significantly inhibited (Figure S1C). The IC50 of paclitaxel was also significantly decreased in the chemoresistant cells after the drug treatment (Figure 2G). As indicated by the flow cytometry analysis, TRAF6 expression, when inhibited, significantly increased the ratio of apoptosis cells under the treatment of paclitaxel (Figure 2H; Figure S1D). All this indicated that TRAF6 promoted the chemoresistance of TNBC cells to paclitaxel.

### 3.3 TRAF6‐promoted paclitaxel resistance in TNBC cells via glycolysis enhanced

**FIGURE 3:**
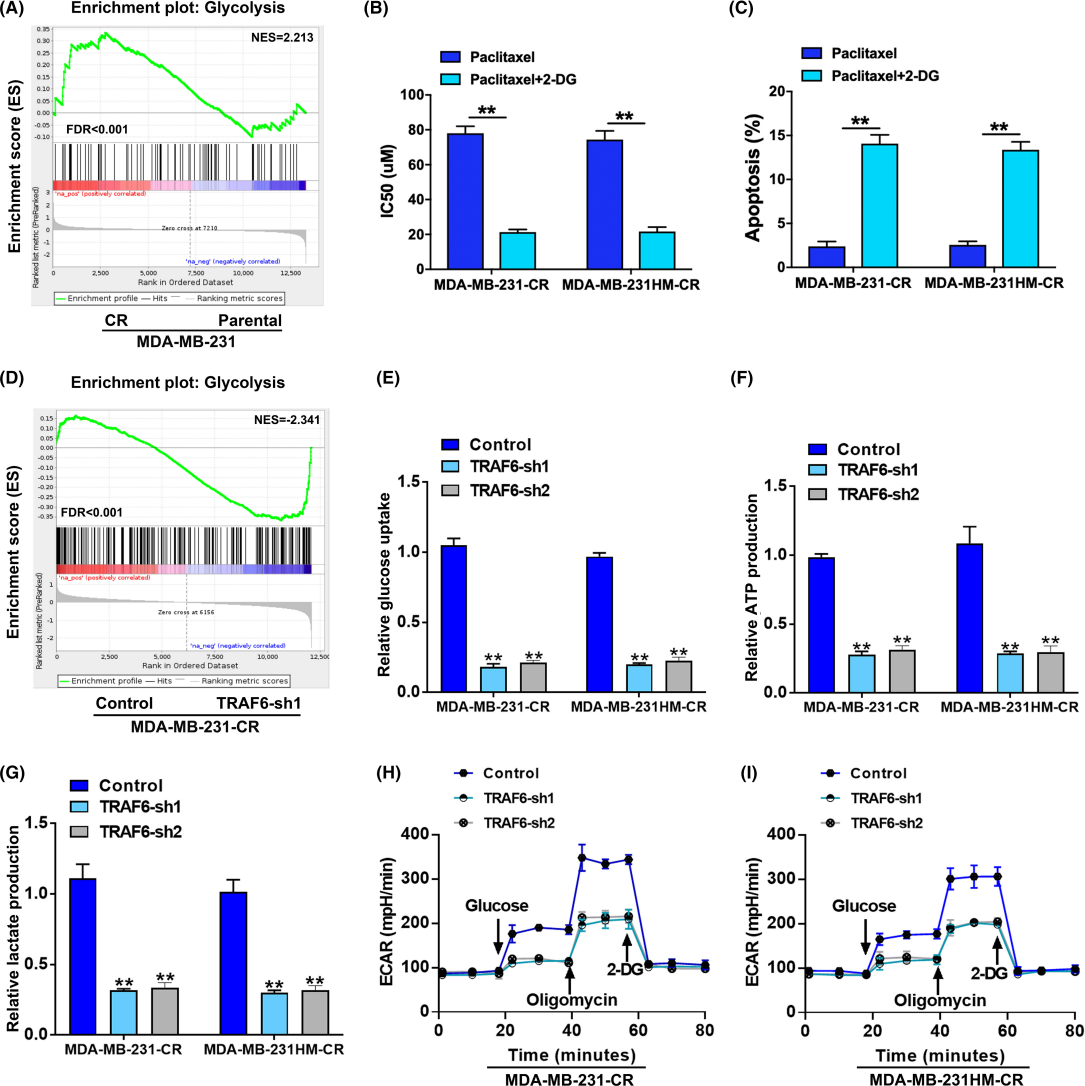
TRAF6‐enhanced glycolysis to facilitate triple‐negative breast cancer (TNBC) chemoresistance. (A) RNA‐sequencing and gene set enrichment analysis (GSEA) showing that glycolysis pathway was activated in TNBC‐CR cells; (B) IC50 values of paclitaxel calculated by CCK‐8 in TNBC‐CR cells with glycolysis inhibited with 2‐DG; (C) paclitaxel‐induced cell apoptosis ratio examined by flow cytometry in TNBC‐CR cells with glycolysis inhibited with 2‐DG; (D) RNA‐sequencing and GSEA indicating that glycolysis pathway was suppressed with TRAF6 expression downregulated; (E–G) glucose uptake, ATP production, and lactate production measured in TNBC‐CR cells with TRAF6 downregulated; (H, I) extra cellular acidification rate (ECAR) monitored in TNBC‐CR cells with TRAF6 expression inhibited; **p < 0.01.

To further explore the mechanism of chemoresistance in TNBC, an analysis was made of the gene expression differences between 231 cells and 231‐CR cells based on RNA‐sequencing; the GSEA showed that the glycolysis metabolic pathway was overactivated in 231‐CR cells compared with 231 cells (Figure 3A). When 2‐Deoxy‐D‐glucose (2‐DG) was added to inhibit glycolysis, the IC50 of paclitaxel was significantly decreased in TNBC‐CR cells (Figure 3B), and the cell apoptosis rate was significantly elevated with paclitaxel treatment (Figure 3C). These findings demonstrated that the chemoresistance of TNBC was positively correlated with the activity of glycolysis.

With the changes in glucose metabolic activity in 231‐CR cells after TRAF6 knockdown by RNA‐sequencing to verify whether TRAF6‐induced drug resistance was correlated with glycolysis, GSEA showed that the inhibited TRAF6 expression suppressed the glycolysis pathway (Figure 3D), which indicated that the downregulated TRAF6 levels reduced glucose uptake (Figure 3E), ATP production (Figure 3F), and lactate generation (Figure 3G). Additionally, the ECAR assay illustrated that the silenced TRAF6 expression restrained the activity of glycolysis in TNBC‐CR cells (Figure 3H,I). These findings revealed that TRAF6 functions to promote the glycolysis‐mediated chemoresistance of TNBC cells.

### 3.4 Glycolysis‐mediated chemoresistance promoted by TRAF6 binding to PKM2

**FIGURE 4:**
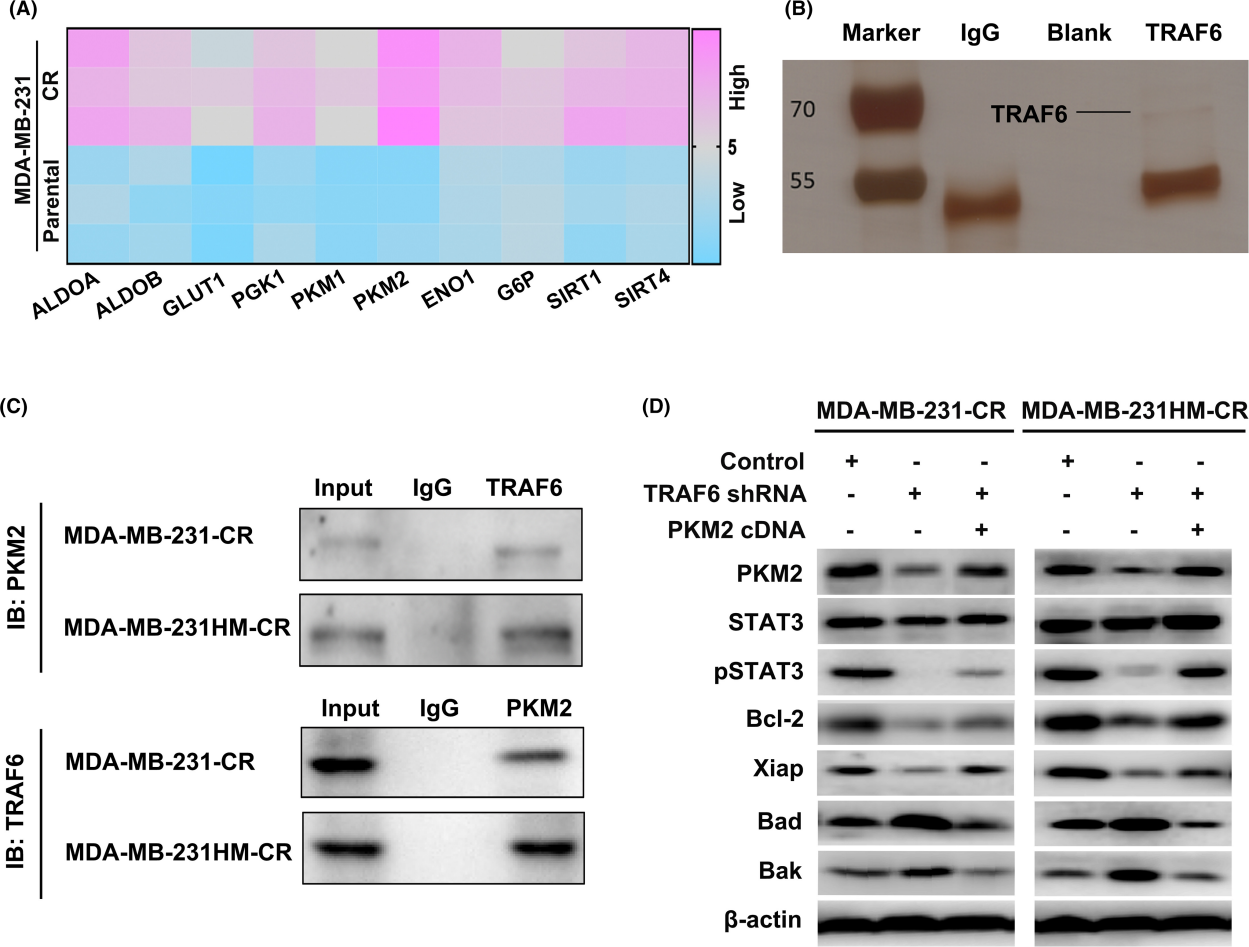
The interaction of TRAF6 with PKM2 in triple‐negative breast cancer (TNBC) cells. (A) qRT‐PCR‐based heatmap showing significant difference in the expression of glycolysis‐related genes between 231 and 231‐CR cells; (B) the binding proteins of TRAF6 identified by immunoprecipitation and silver nitrate staining; (C) Co‐IP and western blotting used to testify the interaction between TRAF6 with PKM2; (D) the expression of the related proteins in TNBC‐CR cells detected by western blotting with TRAF6 downregulated or PKM2 overexpressed.

In the further exploration of the molecular mechanism of TRAF6 regulating glycolysis, qRT‐PCR was performed to examine mRNA expression levels of the key glycolysis‐related enzymes such as ALDOA, ALDOB, GLUT1, PGK1, PKM1, PKM2, ENO1, G6P (glucose‐6‐phosphate), SIRT1, and SIRT4, the results of which revealed that PKM2 was the most significantly upregulated (Figure 4A). Then the immunoprecipitation technique was employed to determine the interacting proteins (Figure 4B), before TRAF6 complex was analyzed by LC/MS, in which 21 binding proteins were identified, one of them being PKM2 (Figure S2A,B). Co‐immunoprecipitation and western blotting further confirmed the interaction between TRAF6 and PKM2 (Figure 4C). These findings indicated that TRAF6 regulated the glycolysis pathway by binding directly to PKM2.

According to the investigation into the biological function of PKM2, in which shRNA was constructed to silence PKM2 expression in the TNBC‐CR cells, the effect was confirmed by western blotting (Figure S3A). When PKM2 level was downregulated, cell viability was significantly inhibited in TNBC‐CR cells after the drug treatment by CCK‐8 (Figure S3B). Additionally, as indicated by the flow cytometry analysis, when PKM2 expression was inhibited, the ratio of apoptosis cells was significantly increased under the treatment of paclitaxel (Figure S3C,D), while the IC50 of paclitaxel decreased significantly in the TNBC‐CR cells after the drug treatment (Figure S3E). In the rescue assays to introduce PKM2 cDNA into TRAF6‐downregulated 231‐CR and 231HM‐CR cells, which were performed to verify our hypothesis that TRAF6 could promote chemoresistance through PKM2, we found that the upregulated PKM2 levels significantly increased the IC50 of paclitaxel (Figure S3F) and decreased the cell apoptosis rate (Figure S3G). Of note were the glucose uptake (Figure S3H), lactate production (Figure S3I), and ECAR (Figure S3J,K), which were significantly elevated after the introduction of PKM2, respectively.

Furthermore, the analysis of western blotting showed that the inhibited of TRAF6 expression in TNBC‐CR cells lowered the levels of PKM2 and anti‐apoptosis proteins of Bcl‐2 and Xiap, respectively, but increased the expression of pro‐apoptosis proteins of Bad and Bak (Figure 4D). However, when overexpressed PKM2 in TRAF6 downregulated 231‐CR and 231HM‐CR cells, the level of apoptosis‐related proteins was reversed (Figure 4D). Furthermore, a significant decrease was observed in the level of phosphorylated STAT3 when TRAF6 expression was inhibited, while there was a significant increase when PKM2 was overexpressed (Figure 4D). Nevertheless, the total STAT3 expression was not significantly altered (Figure 4D). This indicated that TRAF6 promoted the drug resistance of glycolysis‐mediated TNBC cells by binding to PKM2 and phosphorylating STAT3.

### 3.5 TRAF6‐facilitated chemoresistance through PKM2 in vivo

**FIGURE 5:**
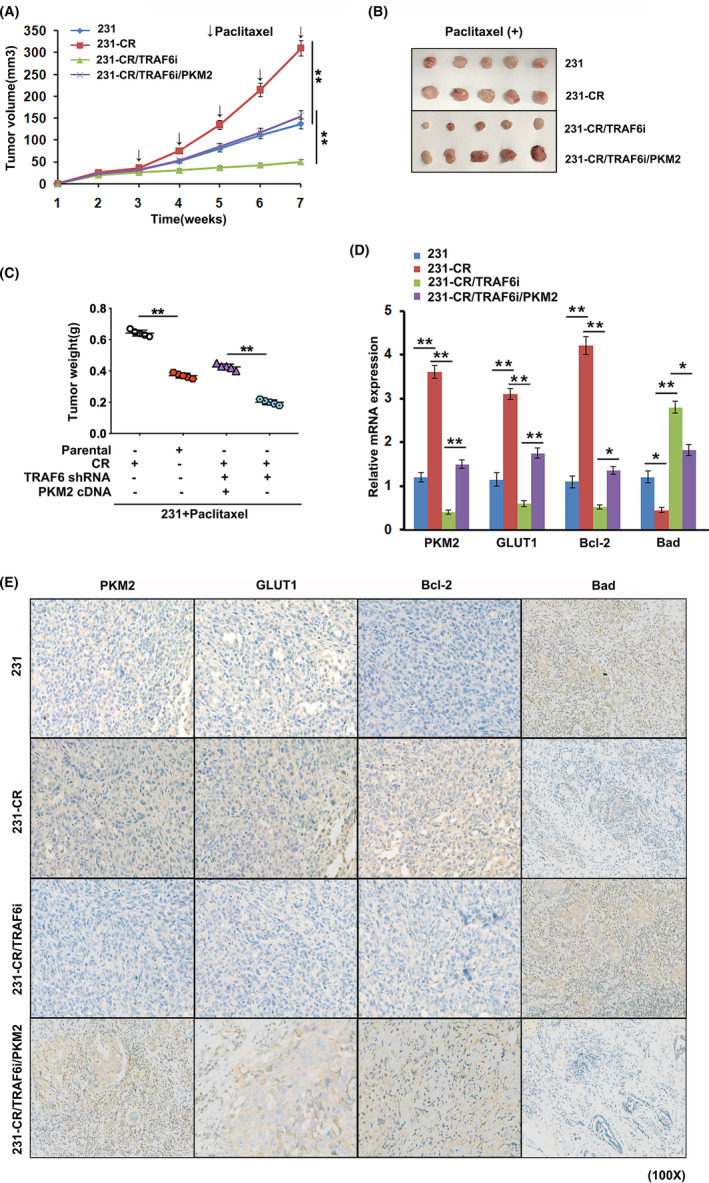
Downregulated TRAF6 to enhance the efficacy of paclitaxel by inhibiting glycolysis in vivo. (A) Tumor growth of the animals injected with 231(MDA‐MB‐231), 231‐CR, 231‐CR/TRAF6i, and 231‐CR/TRAF6i/PKM2 cells to be treated with paclitaxel 3 weeks later; (B) representative images of the tumors dissected; (C) the weight of the tumors analyzed in all groups; (D) relative mRNA levels of glycolysis and apoptosis detected by qRT‐PCR in the related genes; (E) the expression of glycolysis and apoptosis analyzed by immunohistochemistry in the related proteins of the tumor tissues; *p < 0.05, **p < 0.01.

With the establishment of the xenograft models of 231, 231‐CR, 231‐CR/TRAF6i, and 231‐CR/TRAF6i/PKM2 cells to confirm the effect of TRAF6 on TNBC chemoresistance in vivo, the mice were treated with paclitaxel once a week for 3 weeks after inoculation (Figure 5A). The results revealed a marked decrease in tumor volume (Figure 5A,B) and weight (Figure 5C) following the treatment of paclitaxel when TRAF6 expression was suppressed, but a significant increase in tumor volume (Figure 5A,B) and weight (Figure 5C) after PKM2 expression was upregulated. This showed that the inhibited TRAF6 expression enhanced the sensibility of TNBC to paclitaxel in vivo, while the upregulated expression of PKM2 facilitated the regaining of the drug resistance to paclitaxel. Therefore, TRAF6 harbored the ability to promote chemoresistance through PKM2 in TNBC (Figure 5A–C).

From the application of the qRT‐PCR technique, it was found that the expression of PKM2, GLUT1, and Bcl‐2 mRNA significantly increased and that the mRNA expression of Bad significantly decreased in 231‐CR group when compared with the control group (Figure 5D). However, the inhibited TRAF6 expression lowered the levels of PKM2, GLUT1, and Bcl‐2 mRNA and elevated those of Bad mRNA in the TNBC animals, while the upregulated PKM2 expression showed a reversed alteration (Figure 5D). Immunohistochemical assays also revealed that the alterations in the protein level were consistent with those in the mRNA level in all groups (Figure 5E). All this indicated that TRAF6 had the ability to promote TNBC chemoresistance through PKM2 in vivo.

### 3.6 TRAF6‐promoted TNBC chemoresistance through PKM2‐mediated glycolysis in the clinical tumor tissues

**FIGURE 6:**
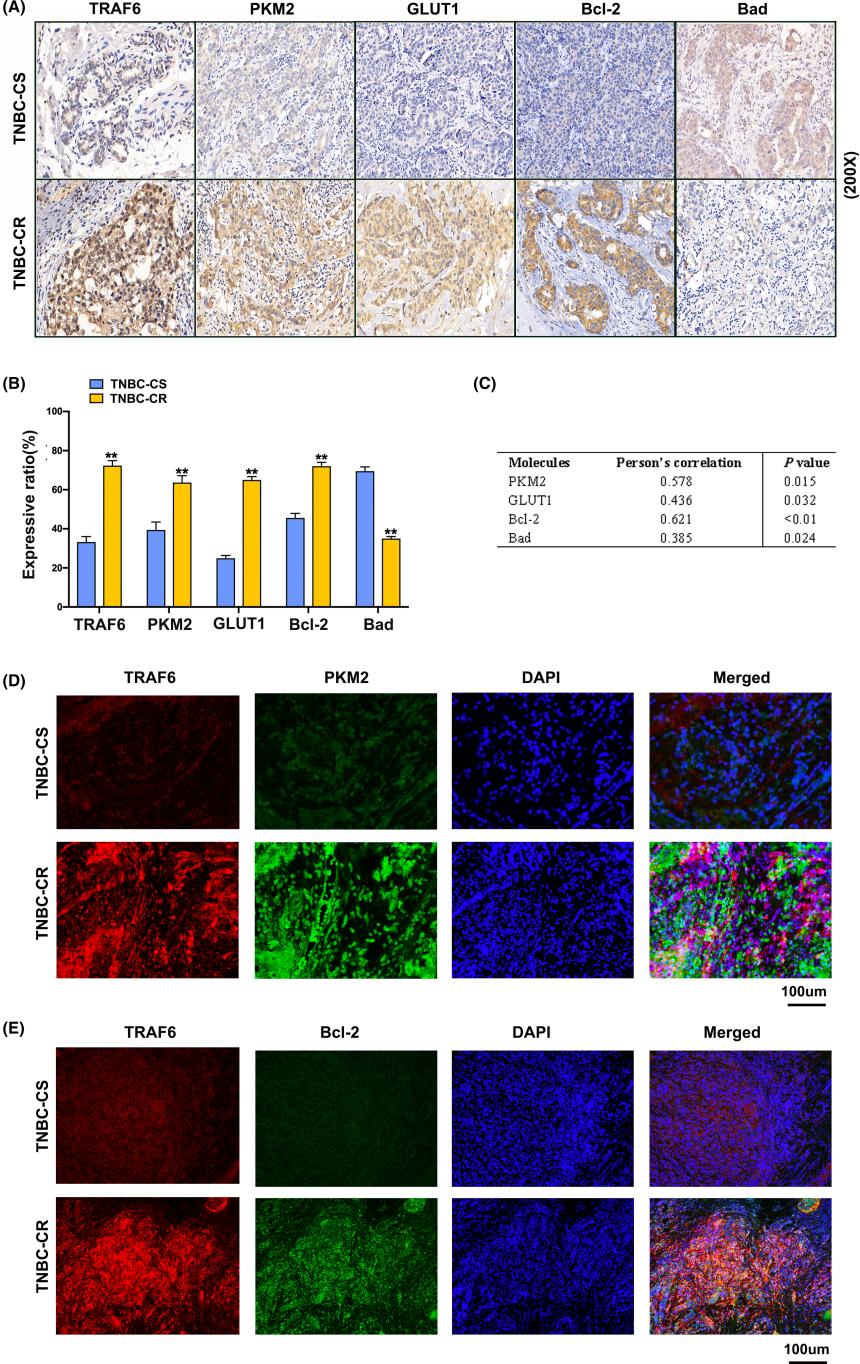
TRAF6‐promoted glycolysis and chemoresistance in the clinical tissues of triple‐negative breast cancer (TNBC). (A) Immunohistochemistry assays of TRAF6, PKM2, GLUT1, Bcl‐2, and Bad expression shown in TNBC‐CR and TNBC‐CS tumor tissues; (B) statistically expressive ratio of TRAF6, PKM2, GLUT1, Bcl‐2, and Bad in TNBC‐CR and TNBC‐CS tumor tissues; (C) correlation analysis of TRAF6 expression with PKM2, GLUT1, Bcl‐2, and Bad expression in TNBC tissues; (D) TRAF6 and PKM2 expression analyzed by immunofluorescence assay in TNBC‐CR and TNBC‐CS tumor tissues; (E) TRAF6 and Bcl‐2 expression analyzed by immunofluorescence assay in TNBC‐CR and TNBC‐CS tumor tissues; **p < 0.01.

Based on the immunohistochemical analysis of 61 chemoresistant (recurrence/metastasis within three post‐operational years) and 124 chemosensitive specimens (non‐recurrence/metastasis within three post‐operational years) from the TNBC patients to examine the clinical correlation of TRAF6 with chemosensitivity, the results revealed that the expression of TRAF6 was significantly higher in the chemoresistant than in the chemosensitive TNBC tissues, which demonstrated a positive correlation of TRAF6 expression with PKM2, GLUT1 and Bcl‐2 expression, and a negative correlation of TRAF6 expression with Bad expression (Figure 6A,B), as shown by the statistical analysis which involved all tumor tissues (Figure 6C). Tissue immunofluorescence assay also confirmed that TRAF6 was correlated with the expressions of PKM2 and Bcl‐2 in chemoresistant TNBC patients, which shared the same location in TNBC tumor tissues (Figure 6D,E). These findings indicated that TRAF6 promoted drug resistance by regulating PKM2 in TNBC patients.

## 4 DISCUSSION

Previously reported studies have shown that TRAF6 is overexpressed in breast cancer,^27^ especially in TNBC patients who produce higher expression than those who carry HR+ and HER2+ breast cancer.^28^ Moreover, TRAF6 has been shown to facilitate tumor proliferation, invasion, and metastasis in multiple types of cancer.^27^, ^29^, ^30^ All this suggests that TRAF6 can function to facilitate the development and progression of TNBC. Although TRAF6 is involved in various axes of regulation to promote tumor chemoresistance,^31^, ^32^, ^33^ there has been a dearth of literature on its role in TNBC chemoresistance. In the current study, we confirmed that TRAF6 was upregulated in TNBC and discovered the downregulation of TRAF6, which could increase the sensitivity to paclitaxel of TNBC cells.

A previously reported study revealed that TRAF6 regulated the PI3K/AKT signal pathway, resulting in the phosphorylation and ubiquitination of AKT and promoting tumor cell growth and proliferation.^34^ MAPKs were also reported to be classical signaling pathways mediated by TRAF6 in tumor progression, ultimately leading to the activation of NF‐κB and AP‐1^10^, ^35^; however, whether TRAF6 performs some other regulatory mechanisms that can contribute to chemoresistance is still worth being explored. Evidence showed that elevated glycolysis was conducted in chemoresistant cancer cells to maintain a higher energy requirement as a result of mitochondrial defects.^36^ Thus, we hypothesized that TRAF6‐promoted drug resistance could be correlated with enhanced glycolysis activation. In the current research, we discovered that the glycolysis pathway was activated in TNBC‐CR cells, that 2‐DG‐inhibited glycolysis increased drug sensitivity, and that downregulated TRAF6 expression significantly facilitated chemotherapeutic effects as the level of glycolysis decreased. Therefore, we concluded that TRAF6 could promote the chemoresistance of TNBC via regulating glycolysis.

Furthermore, we elucidated the specific mechanism by which TRAF6 regulates glycolysis. As a pivotal enzyme in glucose metabolism, PKM2 catalyzes the final rate‐limiting step of glycolysis, involving the conversion of phosphoenolpyruvate and ADP to pyruvate and ATP.^37^ Mounting evidence indicates that inhibiting PKM2 expression could delay tumor progression^22^, ^38^, ^39^ and also increase sensitivity to chemotherapy.^40^, ^41^, ^42^ In our study, we verified that TRAF6 contributed to chemoresistance in TNBC by binding directly to PKM2; that targeting TRAF6 with shRNA downregulated the level of PKM2, enhancing the sensitivity of TNBC to chemotherapeutics in vitro and vivo; and that PKM2 overexpression rescued drug resistance and glycolysis suppressed by TRAF6 depletion in TNBC cells. These findings suggested that PKM2 could act as a key molecule in TRAF6 to promote TNBC resistance to chemotherapy.

Additionally, we proved that inhibited TRAF6 expression lowered the expression of anti‐apoptosis proteins, promoting the expression of pro‐apoptosis proteins, with the phosphorylated level of STAT3 decreased, and that upregulated PKM2 expression significantly increased the level of phosphorylated STAT3 and anti‐apoptosis proteins but reduced the expression of pro‐apoptosis proteins, as manifested by the multiple mechanism research that revealed that PKM2 could translocate into the nuclear to activate STAT3.^43^, ^44^ In view of this, we concluded that TRAF6 could be capable of promoting TNBC chemoresistance by activating PKM2 to activate the STAT3 signal pathway. When TARF6 interacts with PKM2, however, the mechanism by which PKM2 is translocated into the nucleus remains unclear.

In summary, in the current investigation on the function of TRAF6 in TNBC chemoresistance and glucose metabolism, we identified a brand‐new cancer‐driving role of the TRAF6/PKM2/STAT3 axis based on the evidence that TRAF6 interacted with PKM2 to enhance glycolysis and then promote TNBC chemoresistance by activating STAT3 at the level of phosphorylation, and that TRAF6 boosted PKM2‐mediated drug resistance of TNBC in the xenograft models and clinical tumor tissues. Therefore, our findings uncovered the underlying mechanism of TRAF6 to facilitate chemoresistance in TNBC, which can provide a potential therapeutic target for cancer treatment.

## AUTHOR CONTRIBUTIONS

**Han Xu:** Project administration (lead); writing – original draft (lead). **Longzhi Li:** Project administration (supporting). **Bing Dong:** Data curation (supporting). **Ji Lu:** Formal analysis (supporting). **Kun Zhou:** Investigation (supporting). **Xiaoxing Yin:** Conceptualization (equal); writing – review and editing (equal). **Huizhen Sun:** Conceptualization (equal); writing – review and editing (equal).

## CONFLICT OF INTEREST STATEMENT

The authors declare no competing interests.

## Supporting information


Figure S1.

Figure S2.

Figure S3.

Table S1.

## Data Availability

The data that support the findings of this study are available on request from the corresponding author. The data are not publicly available due to privacy or ethical restrictions.

## References

[1] Siegel RL , Miller KD , Fuchs HE , Jemal A . Cancer statistics. CA Cancer J Clin. 2022;72(2022):7‐33. 35020204 10.3322/caac.21708

[2] Kim C , Gao R , Sei E , et al. Chemoresistance evolution in triple‐negative breast cancer delineated by single‐cell sequencing. Cell. 2018;173:879‐893.e813. 29681456 10.1016/j.cell.2018.03.041 PMC6132060

[3] Chaudhary LN , Wilkinson KH , Kong A . Triple‐negative breast cancer: who should receive neoadjuvant chemotherapy? Surg Oncol Clin N Am. 2018;27:141‐153. 29132557 10.1016/j.soc.2017.08.004

[4] Lee A , Djamgoz MBA . Triple negative breast cancer: emerging therapeutic modalities and novel combination therapies. Cancer Treat Rev. 2018;62:110‐122. 29202431 10.1016/j.ctrv.2017.11.003

[5] Mehraj U , Mushtaq U , Mir MA , et al. Chemokines in triple‐negative breast cancer heterogeneity: new challenges for clinical implications. Semin Cancer Biol. 2022;86:769‐783. 10.1016/j.semcancer.2022.03.008 35278636

[6] Feizabadi MS , Castillon VJ . The effect of tau and taxol on polymerization of MCF7 microtubules in vitro. Int J Mol Sci. 2022;23;677‐686. 35054875 10.3390/ijms23020677 PMC8776089

[7] Fremd C , Jaeger D , Schneeweiss A . Targeted and immuno‐biology driven treatment strategies for triple‐negative breast cancer: current knowledge and future perspectives. Expert Rev Anticancer Ther. 2019;19:29‐42. 30351981 10.1080/14737140.2019.1537785

[8] Yang WL , Wang J , Chan CH , et al. The E3 ligase TRAF6 regulates Akt ubiquitination and activation. Science. 2009;325:1134‐1138. 19713527 10.1126/science.1175065 PMC3008763

[9] Huang H , Li X , Yu L , et al. Wogonoside inhibits TNF receptor‐associated factor 6 (TRAF6) mediated‐tumor microenvironment and prognosis of pancreatic cancer. Ann Transl Med. 2021;9:1460. 34734012 10.21037/atm-21-4164 PMC8506702

[10] Kawai T , Akira S . The role of pattern‐recognition receptors in innate immunity: update on toll‐like receptors. Nat Immunol. 2010;11:373‐384. 20404851 10.1038/ni.1863

[11] Nusse R , Clevers H . Wnt/beta‐catenin signaling, disease, and emerging therapeutic modalities. Cell. 2017;169:985‐999. 28575679 10.1016/j.cell.2017.05.016

[12] Meng G , Li G , Yang X , Xiao N . Inhibition of miR146b‐5p suppresses CT‐guided renal cell carcinoma by targeting TRAF6. J Cell Biochem. 2018;120:2382‐2390. 30206978 10.1002/jcb.27566

[13] Guangwei Z , Zhibin C , Qin W , et al. TRAF6 regulates the signaling pathway influencing colorectal cancer function through ubiquitination mechanisms. Cancer Sci. 2022;113:1393‐1405. 35179811 10.1111/cas.15302 PMC8990288

[14] Zhang X , Wu L , Xiao T , et al. TRAF6 regulates EGF‐induced cell transformation and cSCC malignant phenotype through CD147/EGFR. Oncogenesis. 2018;7:17. 29463844 10.1038/s41389-018-0030-1 PMC5833715

[15] Basan M , Hui S , Okano H , et al. Overflow metabolism in Escherichia coli results from efficient proteome allocation. Nature. 2015;528:99‐104. 26632588 10.1038/nature15765 PMC4843128

[16] Beger RD . A review of applications of metabolomics in cancer. Metabolites. 2013;3:552‐574. 24958139 10.3390/metabo3030552 PMC3901293

[17] Semenza GL , Artemov D , Bedi A , et al. ‘The metabolism of tumours’: 70 years later. Novartis Found Symp. 2001;240:251‐260; discussion 260‐254. 11727934

[18] Deberardinis RJ , Sayed N , Ditsworth D , Thompson CB . Brick by brick: metabolism and tumor cell growth. Curr Opin Genet Dev. 2008;18:54‐61. 18387799 10.1016/j.gde.2008.02.003 PMC2476215

[19] Gatenby RA , Gillies RJ . Why do cancers have high aerobic glycolysis? Nat Rev Cancer. 2004;4:891‐899. 15516961 10.1038/nrc1478

[20] Lunt SY , Vander Heiden MG . Aerobic glycolysis: meeting the metabolic requirements of cell proliferation. Annu Rev Cell Dev Biol. 2011;27:441‐464. 21985671 10.1146/annurev-cellbio-092910-154237

[21] Wong N , Ojo D , Yan J , Tang D . PKM2 contributes to cancer metabolism. Cancer Lett. 2015;356:184‐191. 24508027 10.1016/j.canlet.2014.01.031

[22] Christofk HR , Vander Heiden MG , Harris MH , et al. The M2 splice isoform of pyruvate kinase is important for cancer metabolism and tumour growth. Nature. 2008;452:230‐233. 18337823 10.1038/nature06734

[23] Yao X , Li W , Li L , et al. YTHDF1 upregulation mediates hypoxia‐dependent breast cancer growth and metastasis through regulating PKM2 to affect glycolysis. Cell Death Dis. 2022;13:258. 35319018 10.1038/s41419-022-04711-1 PMC8940925

[24] Wang X , Zhang H , Yang H , et al. Exosome‐delivered circRNA promotes glycolysis to induce chemoresistance through the miR‐122‐PKM2 axis in colorectal cancer. Mol Oncol. 2020;14:539‐555. 31901148 10.1002/1878-0261.12629 PMC7053238

[25] Wang X , Zhang F , Wu XR . Inhibition of pyruvate kinase M2 markedly reduces chemoresistance of advanced bladder cancer to cisplatin. Sci Rep. 2017;7:45983. 28378811 10.1038/srep45983 PMC5380992

[26] He Y , Wang Y , Liu H , et al. Pyruvate kinase isoform M2 (PKM2) participates in multiple myeloma cell proliferation, adhesion and chemoresistance. Leuk Res. 2015;39:1428‐1436. 26453405 10.1016/j.leukres.2015.09.019

[27] Shen H , Li L , Yang S , et al. Regulatory role of tumor necrosis factor receptor‐associated factor 6 in breast cancer by activating the protein kinase B/glycogen synthase kinase 3beta signaling pathway. Mol Med Rep. 2017;16:2269‐2273. 28627683 10.3892/mmr.2017.6782

[28] Bilir C , Engin H , Can M , et al. Increased serum tumor necrosis factor receptor‐associated factor‐6 expression in patients with non‐metastatic triple‐negative breast cancer. Oncol Lett. 2015;9:2819‐2824. 26137154 10.3892/ol.2015.3094 PMC4473698

[29] Han F , Zhang L , Qiu W , Yi X . TRAF6 promotes the invasion and metastasis and predicts a poor prognosis in gastric cancer. Pathol Res Pract. 2016;212:31‐37. 26627263 10.1016/j.prp.2015.11.005

[30] Sun H , Li X , Fan L , Wu G , Li M , Fang J . TRAF6 is upregulated in colon cancer and promotes proliferation of colon cancer cells. Int J Biochem Cell Biol. 2014;53:195‐201. 24755241 10.1016/j.biocel.2014.04.010

[31] Meng Q , Liang C , Hua J , et al. A miR‐146a‐5p/TRAF6/NF‐kB p65 axis regulates pancreatic cancer chemoresistance: functional validation and clinical significance. Theranostics. 2020;10:3967‐3979. 32226532 10.7150/thno.40566 PMC7086345

[32] Qian Z , Zhou S , Zhou Z , et al. miR146b5p suppresses glioblastoma cell resistance to temozolomide through targeting TRAF6. Oncol Rep. 2017;38:2941‐2950. 29048680 10.3892/or.2017.5970

[33] Xie C , Zhang LZ , Chen ZL , et al. A hMTR4‐PDIA3P1‐miR‐125/124‐TRAF6 regulatory axis and its function in NF kappa B signaling and chemoresistance. Hepatology. 2020;71:1660‐1677. 31509261 10.1002/hep.30931 PMC7318625

[34] Wang Z , Liu Y , Huang S , Fang M . TRAF6 interacts with and ubiquitinates PIK3CA to enhance PI3K activation. FEBS Lett. 2018;592:1882‐1892. 29729098 10.1002/1873-3468.13080

[35] Wagner EF , Nebreda AR . Signal integration by JNK and p38 MAPK pathways in cancer development. Nat Rev Cancer. 2009;9:537‐549. 19629069 10.1038/nrc2694

[36] Zhou Y , Tozzi F , Chen J , et al. Intracellular ATP levels are a pivotal determinant of chemoresistance in colon cancer cells. Cancer Res. 2012;72:304‐314. 22084398 10.1158/0008-5472.CAN-11-1674 PMC3601736

[37] Bayley JP , Devilee P . The Warburg effect in 2012. Curr Opin Oncol. 2012;24:62‐67. 22123234 10.1097/CCO.0b013e32834deb9e

[38] Goldberg MS , Sharp PA . Pyruvate kinase M2‐specific siRNA induces apoptosis and tumor regression. J Exp Med. 2012;209:217‐224. 22271574 10.1084/jem.20111487 PMC3280873

[39] Spoden GA , Mazurek S , Morandell D , et al. Isotype‐specific inhibitors of the glycolytic key regulator pyruvate kinase subtype M2 moderately decelerate tumor cell proliferation. Int J Cancer. 2008;123:312‐321. 18425820 10.1002/ijc.23512

[40] Li Q , Zhang D , Chen X , et al. Nuclear PKM2 contributes to gefitinib resistance via upregulation of STAT3 activation in colorectal cancer. Sci Rep. 2015;5:16082. 26542452 10.1038/srep16082 PMC4635355

[41] Guo W , Zhang Y , Chen T , et al. Efficacy of RNAi targeting of pyruvate kinase M2 combined with cisplatin in a lung cancer model. J Cancer Res Clin Oncol. 2011;137:65‐72. 20336315 10.1007/s00432-010-0860-5 PMC11827957

[42] Tian S , Li P , Sheng S , Jin X . Upregulation of pyruvate kinase M2 expression by fatty acid synthase contributes to gemcitabine resistance in pancreatic cancer. Oncol Lett. 2018;15:2211‐2217. 29434927 10.3892/ol.2017.7598 PMC5777098

[43] Dhanesha N , Patel RB , Doddapattar P , et al. PKM2 promotes neutrophil activation and cerebral thromboinflammation: therapeutic implications for ischemic stroke. Blood. 2022;139:1234‐1245. 34529778 10.1182/blood.2021012322 PMC8874361

[44] Damasceno LEA , Prado DS , Veras FP , et al. PKM2 promotes Th17 cell differentiation and autoimmune inflammation by fine‐tuning STAT3 activation. J Exp Med. 2020;217; e20190613. 32697823 10.1084/jem.20190613 PMC7537396

