# Supplementary figures and images for: TRAF6 promotes chemoresistance to paclitaxel of triple negative breast cancer via regulating PKM2‐mediated glycolysis
Source: Cancer Med. 2023 Sep 25;12(19):19807–20. doi: 10.1002/cam4.6552 (PMC10587986; doi:10.1002/cam4.6552)

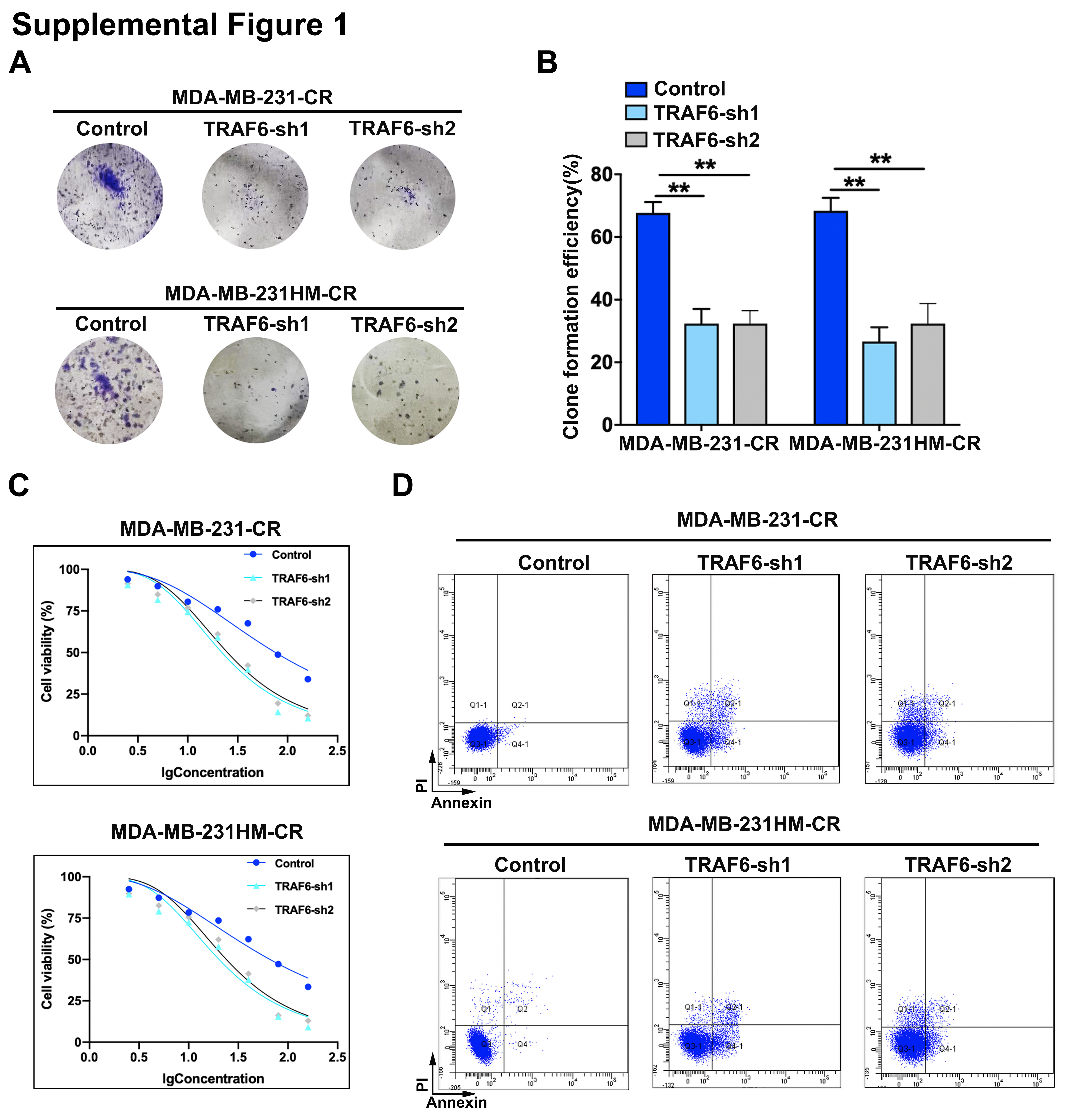

Supplement: Supplementary file 1 — Figure S1. [file CAM4-12-19807-s004.jpg]

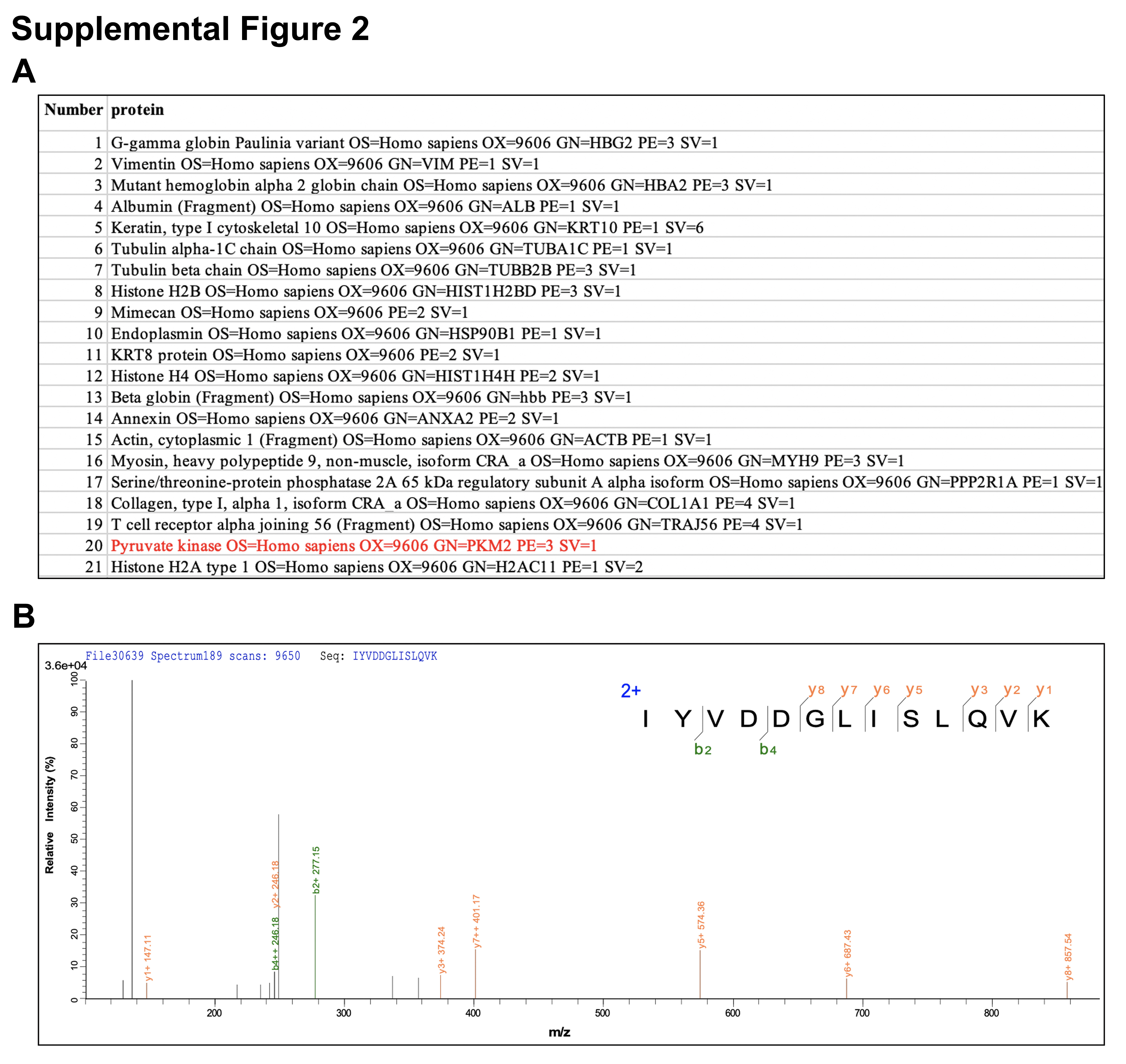

Supplement: Supplementary file 2 — Figure S2. [file CAM4-12-19807-s003.jpg]

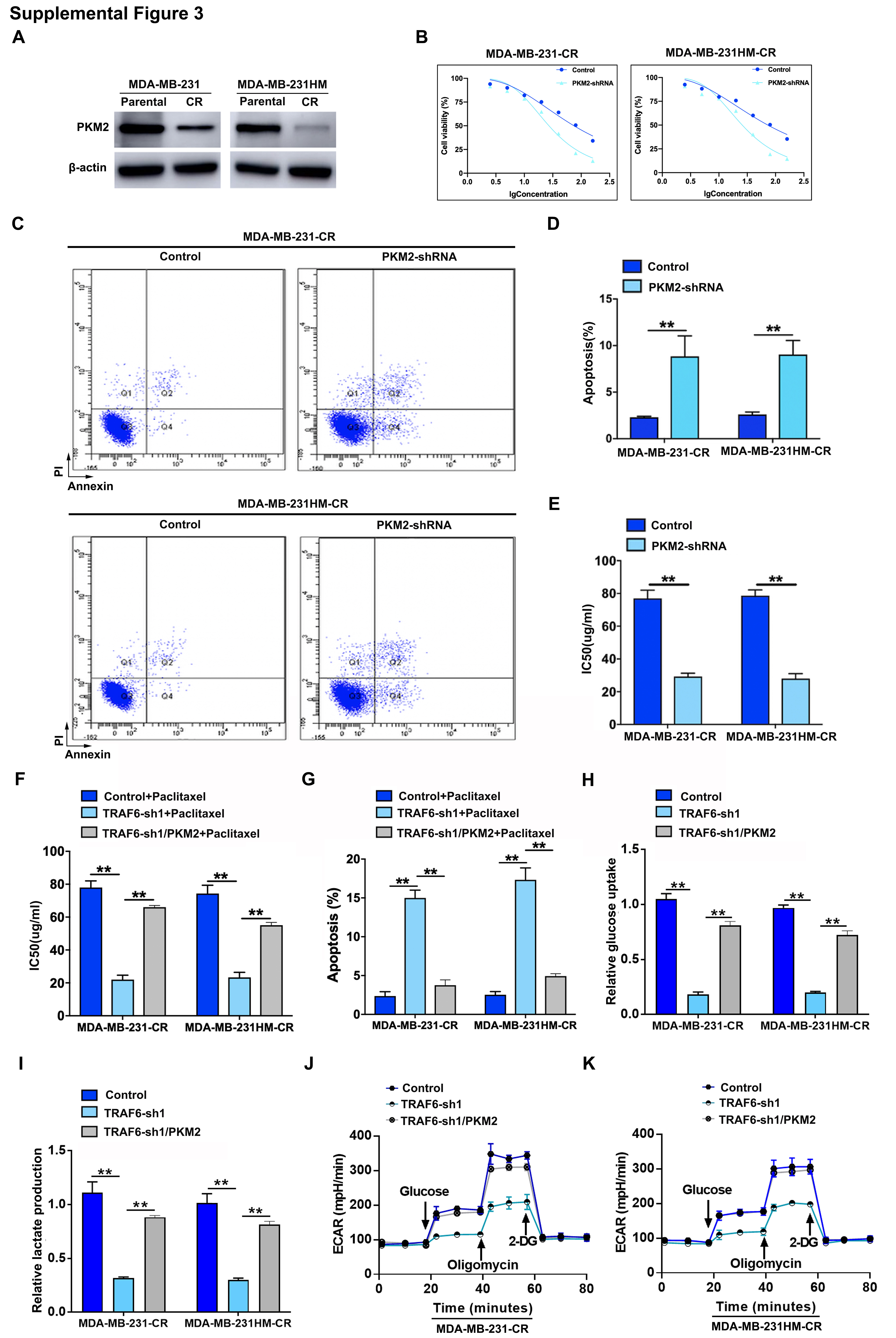

Supplement: Supplementary file 3 — Figure S3. [file CAM4-12-19807-s002.jpg]
