# Supplementary material for: TRAF6 promotes chemoresistance to paclitaxel of triple negative breast cancer via regulating PKM2‐mediated glycolysis
Source: Cancer Med. 2023 Sep 25;12(19):19807–20. doi: 10.1002/cam4.6552 (PMC10587986; doi:10.1002/cam4.6552)
Supplement: Supplementary file 4 — Table S1. [file CAM4-12-19807-s001.docx]

**Supplemental Table 1** Clinicalpathological characteristics of patients and univariate analysis of correlation between related factors expression with chemoresistance in TNBC

| Variables | Chemosensitive（CS） | | Chemoresistant（CR） | | P value |
| --- | --- | --- | --- | --- | --- |
|  | NO | Percent(%) | NO | Percent(%) |  |
| Age |  |  |  |  |  |
| <=50 | 60 | 48.4 | 29 | 47.5 | 0.806 |
| >50 | 64 | 51.6 | 32 | 52.5 |  |
| Tumor Size |  |  |  |  |  |
| <=2cm | 54 | 43.5 | 24 | 39.3 | 0.524 |
| >2cm | 70 | 56.5 | 37 | 60.7 |  |
| Histological grade |  |  |  |  |  |
| I-II | 93 | 75.0 | 38 | 62.3 | 0.215 |
| III | 31 | 25.0 | 23 | 37.7 |  |
| Lymph node M |  |  |  |  |  |
| Negative | 76 | 61.3 | 31 | 50.8 | 0.137 |
| Positive | 48 | 38.7 | 30 | 49.2 |  |
| TRAF6 expression |  |  |  |  |  |
| High | 40 | 32.3 | 44 | 72.1 | 0.005 |
| Low | 84 | 67.7 | 17 | 27.9 |  |
| PKM2 expression |  |  |  |  |  |
| High | 48 | 38.7 | 39 | 63.9 | 0.015 |
| Low | 76 | 61.3 | 22 | 26.1 |  |
| GLUT1 expression |  |  |  |  |  |
| High | 29 | 23.4 | 40 | 65.6 | 0.003 |
| Low | 93 | 76.6 | 19 | 34.4 |  |
| Bcl-2 expression |  |  |  |  |  |
| High | 54 | 43.5 | 44 | 72.1 | 0.023 |
| Low | 70 | 56.5 | 17 | 27.9 |  |
| Bad expression |  |  |  |  |  |
| High | 86 | 69.4 | 21 | 34.4 | 0.006 |
| Low | 36 | 30.6 | 40 | 65.6 |  |
